# A Transparent Teleoperated Robotic Surgical System with Predictive Haptic Feedback and Force Modelling

**DOI:** 10.3390/s22249770

**Published:** 2022-12-13

**Authors:** Taran Batty, Armin Ehrampoosh, Bijan Shirinzadeh, Yongmin Zhong, Julian Smith

**Affiliations:** 1 Australian Synchrotron, ANSTO, Melbourne, VIC 3168, Australia; 2 Robotics and Mechatronics Research Laboratory (RMRL), Department of Mechanical and Aerospace Engineering, Monash University, Melbourne, VIC 3800, Australia; 3 Department of Mechanical and Automotive Engineering, RMIT University, Melbourne, VIC 3083, Australia; 4 Department of Surgery, Monash University, Melbourne, VIC 3800, Australia

**Keywords:** teleoperated robotic surgery, medical robotics, haptic feedback, predictive force feedback, force modelling

## Abstract

In recent years, robotic minimally invasive surgery has transformed many types of surgical procedures and improved their outcomes. Implementing effective haptic feedback into a teleoperated robotic surgical system presents a significant challenge due to the trade-off between transparency and stability caused by system communication time delays. In this paper, these time delays are mitigated by implementing an environment estimation and force prediction methodology into an experimental robotic minimally invasive surgical system. At the slave, an exponentially weighted recursive least squares (EWRLS) algorithm estimates the respective parameters of the Kelvin–Voigt (KV) and Hunt–Crossley (HC) force models. The master then provides force feedback by interacting with a virtual environment via the estimated parameters. Palpation experiments were conducted with the slave in contact with polyurethane foam during human-in-the-loop teleoperation. The experimental results indicated that the prediction RMSE of error between predicted master force feedback and measured slave force was reduced to 0.076 N for the Hunt–Crossley virtual environment, compared to 0.356 N for the Kelvin–Voigt virtual environment and 0.560 N for the direct force feedback methodology. The results also demonstrated that the HC force model is well suited to provide accurate haptic feedback, particularly when there is a delay between the master and slave kinematics. Furthermore, a haptic feedback approach that incorporates environment estimation and force prediction improve transparency during teleoperation. In conclusion, the proposed bilateral master–slave robotic system has the potential to provide transparent and stable haptic feedback to the surgeon in surgical robotics procedures.

## 1. Introduction

Master–slave teleoperated robotic systems are being applied to a wide range of fields, from the deep sea to space exploration and from mining to medical industries. Robotic minimally invasive surgery (RMIS) is one such field benefiting from the improved levels of control offered by teleoperated systems. Teleoperated robotics are capable of delivering improvements in motion scaling, hand tremor filtering, and hand-eye co-ordination, as well as enabling the automation of specific quantifiable actions, such as suturing [1,2,3,4].

Bilateral teleoperators are teleoperated systems capable of providing force feedback or haptic feedback to the operator [5,6]. Presently, commercial RMIS systems do not include haptic feedback [7,8], which has been reported as one of the disadvantages associated with RMIS systems [9,10,11,12]. The lack of an intuitive force feedback mechanism contributes to the technique’s steep learning curve and slow completion time.

The difficulty in providing haptic feedback for surgical applications stems from the fact that traditional bilateral controllers are unable to provide high levels of transparency and stability simultaneously [13]. Ideal or perfect transparency is defined as the matching between the impedance felt by the operator and that experienced at the slave. Typically, this is achieved via kinematic and force correspondence between slave and master subsystems. First realised in [14] by Lawrence and extended in [15] by Hashtrudi and Salcudean with the inclusion of modelled time delays, transparency and stability have been shown to occupy opposing ends of a spectrum. Improving transparency degrades stability while increasing stability mutes system transparency [16,17,18]. Additionally, perfect transparency is not possible in traditional feedback/feedforward bilateral controllers due to this innate communication time delay between slave and master subsystems [19].

The literature contains a wide range of algorithms and approaches designed to address the issue of communication time delay in bilateral teleoperations. The majority of these approaches are based on the passivity control theory [20,21]. Using this approach, the communication network, which is a source of non-passivity among the other teleoperation control subsystems (including the operator and environment, both of which are considered passive components), is transformed into a passive element, resulting in a stable and passive system as a whole. Among the most commonly used passivity-based techniques is wave variable control (WVC) [22,23], which involves transforming force and motion into wave variables, resulting in the passivity of the communication network being limited to an unknown and constant delay. There is also the time domain passivity control (TDPC) [24] technique that dissipates the additional and nonpassive energy by injecting adaptive damping into the system in the event that there is an unknown and variable time delay. As an alternative to passivity-based control, there is the small gain control theory [25], which does not require the assumption of passivity of system components since it deals with the overall loop gain of the system. However, all of the algorithms above alter the flow of force and motion data in some form in order to mitigate the excess energy that adversely affects the system’s transparency and results in an infeasible haptic feedback system for surgical applications [13].

These restrictions have prompted the use of predictive control in place of the approaches mentioned above and direct force feedback methodologies [26,27,28,29]. Predictive control strategies are able to compensate for the system time delay by using local models at the master and/or slave to generate operator feedback forces. A majority of these predictive controllers use Smith predictors [30,31,32,33] and neural networks [34,35,36] to compensate for communication time delays. An overview of the various predictive control approaches can be found in [30].

A similar predictive approach involves modelling the environment on-line, using estimation methods such as the Recursive Least Squares (RLS) or Exponentially Weighted Recursive Least Squares (EWRLS) [37,38,39]. By developing an estimation of the environment, a virtual environment can be created with which the haptic device can interact. Force-feedback can then be tied to the operator’s own hand movements, effectively bypassing the time delay present between the slave and master subsystems and the need to wait for the slave system to measure the response. Further, provided the estimated parameters are a good representation of the environment, then stability is guaranteed under the assumption that the contact environment is passive.

The Kelvin–Voigt (KV) force model, consisting of the parallel connection of a linear spring and damper, has been widely used as an underlying environment force model [40,41]. However, the KV model has several inconsistencies in relation to power exchange and restitution during contact [42], which makes it unsuitable for modelling for soft-body contact. Further, the Kelvin–Voigt model is a linear model, whereas most biological soft tissues are non-linear [43,44].

The Hunt–Crossley (HC) force model demonstrates more consistency with the dynamic behaviour of soft bodies [42]. Palpation exercises conducted in [45], comparing a number of different force models, found that the Hunt–Crossley model demonstrated superior distinction between regions of varying stiffness. However, human-in-the-loop operation was not evaluated, and force feedback was limited to a position–position architecture. Estimating the parameters of the Hunt–Crossley force model has been previously conducted by partially decoupling the estimation of the model parameters via separate processes [42], which results in relatively slow parameter convergence. In [46], a single-staged identification method was developed whereby the Hunt–Crossley model was linearised via a logarithmic approximation. With the log-linearised form, parameter estimation could then be conducted using the robust family of RLS algorithms. In [47], parameter estimation was conducted on-line and demonstrated comparatively faster parameter convergence when compared to the two-stage identification process. However, these estimations were performed with a computer-generated trajectory and did not include human-in-the-loop teleoperation.

In cases where remote environments included materials with different rigidity, the researchers also investigated a hybrid approach using a combination of KV and HC models called the threshold contact switching model (TSCM) [48]. For adapting to different contact environments, a threshold was used to switch between each model. While this approach had some advantages, it was limited by undesired inconsistency and oscillation when switching between different models. To resolve this issue, a continuous switching contact model (CSCM) based on energy loss was developed [49,50,51]. However, further improvements are still required in terms of accuracy and transparency.

Human-in-the-loop teleoperation presents additional challenges, particularly when an estimation of the environment is desired. When the slave mechanism’s motion is driven by a human-controlled device (i.e., a haptic-feedback device), the question of persistency of excitation becomes important. Does the environment estimator receive enough varying information (force, position, or velocity) to accurately estimate the contact environment’s parameters? Additionally, the feedforward/feedback nature of the bilateral controller means that teleoperator performance needs to be evaluated holistically. The goal of this work is to experimentally verify the performance of an on-line environment estimation–force prediction methodology; to evaluate teleoperator performance as a whole. It also aims to demonstrate that transparent teleoperation can be achieved with an estimated virtual environment, which is interacted with by the haptic master device.

The main contributions of this research are as follows: first, development, characterisation, and experimental validation of a high-transparency predictive force-feedback methodology. In this study, an environment estimation–force prediction control architecture is developed and implemented into a three-channel bilateral teleoperated system. The use of a force predictor means that neither kinematic nor force correspondence is required to be strictly maintained. Secondly, investigation of the human-in-the-loop teleoperation transparency performance of the bilateral robotic system by studying various force modelling approaches in isolation, including a spring-based adaptor, KV, and HC force models. Lastly, a metric for transparency is developed that accounts for the positional lag between slave and master systems. By using the current slave position as a reference, and comparing forces at a prior comparable master position, an accurate measure of error can be determined on the fly.

The remainder of this paper is organised as follows: Section 2 describes an overview of the proposed system the environment models chosen, and the parametrisation method used for each sample. Section 3 outlines the metric used to evaluate the transparency performance. Section 4 describes the experimental platform and procedure. Section 5 presents the experimental results of a palpation exercise into soft polyurethane foam. Finally, Section 6 presents the conclusions and suggestions for future work.

## 2. Environment Estimation and Force Prediction Controller

**Figure 1:**
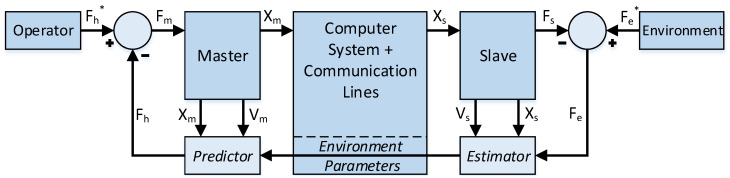
Environment estimation and force prediction controller overview.

An overview of the proposed control structure for the estimation–prediction-based force feedback methodology is presented in Figure 1. The estimator relies on the choice of a force model that can accurately describe the environmental response, as well as a robust estimation algorithm to quantify this description into constituent environment parameters. The predictor is subsequently able to use these parameters to generate a feedback force relative to the master kinematics. In essence, the process creates a virtual representation of the environment, effectively predicting the force response given a set of kinematic values.

### 2.1. Kelvin–Voigt Force Model

The Kelvin–Voigt (KV) linear contact model (1) is a common force model used in robotic systems in the modelling of soft-body contact. It is a linear combination of a spring-damper system, and is widely used as it allows simple and robust parameter estimation. The force equation is

(1) $$F_{e}\left(t\right)=\left\{\begin{array}{cc} K_{KV}x\left(t\right)+B_{KV}\dot{x}\left(t\right), & x(t)\geq 0\\ 0 & x(t)<0 \end{array}\right.$$

where x(t) is the penetration depth inside the contact environment, $\dot{x}\left(t\right)$ is the velocity, and the parameters $B_{KV}$ and $K_{KV}$ represent the stiffness and damping coefficients, respectively.

The KV model has several physical inconsistencies that make it non-ideal for modelling soft-contact environments. A non-zero velocity at the boundary (i.e., $F_{e}\neq 0$ when $\left|x\right|=0$ and $\left|\dot{x}\right|>0$) results in a non-zero contact force. This translates to a ‘shock’ force during insertion and a ‘sticking’ feeling during retraction. More specifically, power exchange between tool and environment is inconsistent with physical reality. Additionally, the majority of biological soft tissues are non-linear, and a linear model such as the Kelvin–Voigt model may be ill -uited to predict the behaviour of such contact environments.

### 2.2. Hunt–Crossley Force Model

The Hunt–Crossley force model (2) attempts to provide a better model of soft-body contact by including a position dependence into the damping term. Additionally, non-linearity is included in the position term to model non-linear behaviour reported in many biological tissues. The Hunt–Crossley force model can be defined as:

(2) $$F_{e}\left(t\right)=\left\{\begin{array}{cc} K_{HC}x^{n}\left(t\right)+B_{HC}x^{n}\left(t\right)\dot{x}\left(t\right), & x(t)\geq 0\\ 0 & x(t)<0. \end{array}\right.$$

where *x* is the position, and $\dot{x}$ is the velocity of the end-effector inside the environment; $K_{HC}$, $B_{HC}$, and *n* are the environment stiffness, damping, and non-linearity parameters, respectively; and $F_{e}$ is the tool-environment contact force.

The non-linearity of (2) means that traditional methods to resolve the parameters $K_{HC}$, $B_{HC}$, and *n* are not applicable, as most on-line parameter estimation techniques require a linear system model. Instead, a linearised approximation of the Hunt–Crossley force model is used during the estimation process [46]:

(3) $$\begin{array}{c} \ln\left[F_{e}\right]≅\ln\left(K_{HC}\right)+\frac{B_{HC}}{K_{HC}}{\dot{x}}_{s}\left(t\right)+n\ln\left[x_{s}\left(t\right)\right]+\frac{\epsilon }{K_{HC}x_{s}^{n}\left(t\right)}. \end{array}$$

The derivation of (3) can be found in [46]. Here, the subscript (*s*) indicates the use of the slave-specific quantities. The error term, ϵ, includes the modelling error that arises from the log-linearised form, as well as sensor noise. For this research, ϵ is assumed to be negligible to the estimation process.

### 2.3. Environment Estimator

The linearised force models are parametrised in the form of (4), where parameter estimation can be accomplished through a recursive least-squares (RLS) approach.

(4) $$y_{k}=\phi_{k}^{T}\theta_{k}+\epsilon_{k}$$

Here, $\phi_{k}^{T}$ is the regressor vector, $\theta_{k}$ is the vector of dynamic parameters, and $\epsilon_{k}$ is the modelling error and sensor noise, with each vector being for time-step *k*. The linearised vectors to (4) are shown in Table 1.

The RLS methods are a powerful family of computational tools used to recursively estimate system parameters by minimising a least squares cost function relating to the input signals. The exponentially weighted recursive least squares (EWRLS) algorithm is particularly useful for environments with varying parameters, as the relative weight that past estimates have on the current estimate can be specified and adjusted as necessary. The EWRLS update algorithm is defined as:

(5a) $$\begin{array}{cc} L_{k+1} & =\frac{P_{k}\phi_{k+1}}{\lambda +\phi_{k+1}^{T}P_{k}\phi_{k+1}} \end{array}$$

(5b) $$\begin{array}{cc} P_{k+1} & =\frac{1}{\lambda }\left[P_{k}-L_{k+1}\phi_{k+1}^{T}P_{k}\right] \end{array}$$

(5c) $$\begin{array}{cc} {\hat{\theta }}_{k+1} & ={\hat{\theta }}_{k}+L_{k+1}\left[y_{k+1}-\phi_{k+1}^{T}{\hat{\theta }}_{k}\right]. \end{array}$$

Here, *P* is the covariant matrix, and λ is the forgetting factor. The forgetting factor (λ) acts to minimise the weighting of past inputs during the estimation process. Typically 0.98<λ≤1.0. A forgetting factor of λ=1 resolves (5) into the RLS algorithm, which takes into account all previous data points equally. For the experiments conducted, a static forgetting factor of λ=0.995 was used, which was found to offer a good compromise between estimator speed and stability.

### 2.4. Force Feedback and Force Predictor

Using the predictive force approach, the EWRLS algorithm uses the measured slave dynamics ($F_{e}$, $x_{s}$, and ${\dot{x}}_{s}$) at each time-step to resolve an estimate for the environment parameters of the specified force model. These parameters are then used to create a virtual environment with which the haptic device can interact. Using the Hunt–Crossley model, the force feedback at each time-step [k] is calculated according to

(6) $$F_{m}\left[k\right]=\left\{\begin{array}{cc} {\hat{K}}_{HC}x_{m}^{\hat{n}}\left[k\right]+{\hat{B}}_{HC}x_{m}^{\hat{n}}\left[k\right]{\dot{x}}_{m}\left[k\right], & x_{m}\left[k\right]\geq 0\\ 0 & x_{m}\left[k\right]<0. \end{array}\right.$$

In (6), the estimated environment parameters ${\hat{K}}_{HC}$ and ${\hat{B}}_{HC}$ represent the spring- and damping-like coefficients, respectively, while $\hat{n}$ is the non-linearity coefficient. Equation (6) uses the haptic stylus kinematics ($x_{m}\left[k\right]$ and ${\dot{x}}_{m}\left[k\right]$) and estimated environment parameters (${\hat{K}}_{HC},{\hat{B}}_{HC}$ and $\hat{n}$) to resolve the feedback force, $F_{m}\left[k\right]$.

The same process is used for the Kelvin–Voigt force model, albeit with the Kelvin–Voigt force equation used as the basis. By estimating the environment with the Kelvin–Voigt force model, force feedback can be driven via an entirely virtual environment. In the following, force feedback is calculated according to

(7) $$F_{m}\left[k\right]=\left\{\begin{array}{cc} {\hat{K}}_{KV}x_{m}\left[k\right]+{\hat{B}}_{KV}{\dot{x}}_{m}\left[k\right], & x_{m}\left[k\right]\geq 0\\ 0 & x_{m}\left[k\right]<0. \end{array}\right.$$

where ${\hat{K}}_{KV}$ and ${\hat{B}}_{KV}$ represent the estimated stiffness and damping coefficients of the environment, respectively. Provided the models are valid for the environment, and the estimated parameters converge to the true environment parameters, then the virtual environment can confidently be used to provide transparent haptic feedback.

In order to demonstrate the effectiveness of the predictive force feedback methodology in comparison with other traditional force control methods, two conventional feedback force calculation methods, direct force feedback and spring-based adaptors were also experimentally implemented.

A direct force-feedback controller is examined, whereby at time-step *k*,

(8) $$F_{m}\left[k\right]=F_{s}\left[k\right].$$

The spring-based adaptor implemented into this teleoperated system in [52] places a spring *between* the master and the slave to adapt the feedback force. This technique is a *semi-* or *quasi-direct* feedback methodology, as the underlying force-feedback mechanism is still the environment force, with the spring estimator adapting the final feedback force. Force feedback, in this case, is given by

(9) $$F_{m}\left[k\right]=F_{s}\left[k\right]+{\hat{K}}_{1}\left[k\right]\left(x_{m}\left[k\right]-x_{s}\left[k\right]\right)$$

where the spring-adaptor, ${\hat{K}}_{1}\left[k\right]$, is calculated at each time-step by

(10) $${\hat{K}}_{1}\left[k\right]=\left|\frac{F_{s}\left[k\right]-F_{contact}}{x_{s}\left[k\right]-x_{contact}}\right|.$$

Equation (10) calculates the average stiffness given the slave’s current penetration into the environment ($x_{s}-x_{contact}$) and the difference in force ($F_{s}\left[k\right]-F_{contact}$).

## 3. Transparency Performance

**Figure 2:**
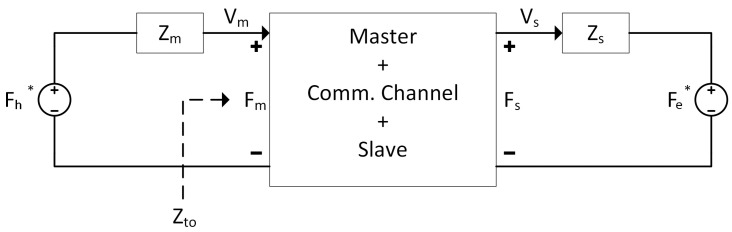
Equivalent circuit diagram of a bilateral teleoperated system.

Figure 2 represents an equivalent circuit diagram of the bilateral teleoperator system shown in Figure 1. Here, *V*, *F*, and *Z* refer to velocity, force, and impedance, respectively. The subscript *s* and *m* refer to the slave and master, respectively. In this figure, $F_{h}^{*}$ is the operator’s force, and $F_{e}^{*}$ is the environment’s exogenous input force. $F_{m}$ is the master feedback, and $F_{s}$ is the measured slave force. $Z_{m}$ and $Z_{s}$ are impedances representing the dynamics of the operator’s hand and remote environment, respectively. $V_{m}$ is the master velocity, and $V_{s}$ is the slave velocity. $Z_{to}$ is the impedance perceived by the operator. Impedance, *Z*, encompasses physical mass, damping, and stiffness properties, and each quantity is the Laplace transform of their respective variable. It is generally assumed that the operator and environment are passive (and thus stable), as they do not act in such a way as to produce or inject additional energy into the system; thus, $F_{h}^{*}=0$ and $F_{e}^{*}=0$ [53].

The linear time-invariant (LTI) dynamics of the above system are:

(11a) $$\begin{array}{cc} F_{m} & =F_{h}^{*}-Z_{h}V_{h} \end{array}$$

(11b) $$\begin{array}{cc} F_{s} & =F_{e}^{*}+Z_{e}V_{e}. \end{array}$$

The impedance experienced by the operator is defined as:

(12) $$Z_{to}=\frac{F_{m}}{V_{m}}{|}_{F_{e}^{*}=0}.$$

Lawrence [14] defines the transparency condition as:

(13) $$Z_{to}=Z_{s}.$$

Equation (13) translates to the operator ($Z_{to}$) experiencing the same environmental behaviour as the slave ($Z_{s}$).

This approach is known as *impedance matching* and is a consequence of the sought-after *kinematic correspondence* (14a) and *force reflection* (14b) between the master and the slave:

(14a) $$\begin{array}{cc} V_{s} & =V_{m} \end{array}$$

(14b) $$\begin{array}{cc} F_{m} & =F_{s}. \end{array}$$

With these definitions, a *hybrid* matrix can be developed, as shown below:

(15) $$\left[\begin{array}{c} F_{m}\\ -V_{s} \end{array}\right]=\left[\begin{array}{cc} h_{11} & h_{12}\\ h_{21} & h_{22} \end{array}\right]\left[\begin{array}{c} V_{m}\\ F_{s} \end{array}\right]$$

Using the kinematic and force correspondence conditions in (14a,b), and the hybrid matrix of (15), the *ideal* transparency is defined as (with no force or position scaling):

(16) $$H_{ideal}=\left[\begin{array}{cc} 0 & 1\\ -1 & 0 \end{array}\right]$$

The proposed bilateral controller has two definitions of error that can be used to evaluate teleoperator transparency performance: estimation error and prediction error, each with a definition describing the accuracy of different processes. The estimation error is localised to the slave–environment interaction and indicates the accuracy of the estimated parameters in reference to the measured forces at the slave. The prediction error relates the interaction between the master and virtual environment to the interaction of the slave with the measured environment. The prediction error encompasses the master’s dynamics, which typically extends beyond the range of the slave–environment dynamics. As such, the prediction error provides an indication of system transparency, whereas the estimation error indicates estimator transparency.

### 3.1. Prediction Error

Prediction error is the error between the predicted master force and the measured slave forces at a given position inside the real and virtual environments. It is used as a metric for transparency, given that it evaluates how well the predictive force feedback mechanism is operating in terms of the teleoperation system as a whole.

A method for calculating the prediction error involves comparing the measured current force with the predicted master force at an unknown time in the past. This is because, at each time-step, the slave and the master are typically at different positions within the real or virtual environment and, thus, are expected to experience different forces and cannot be compared directly. Instead, the forces are compared when the slave and the master kinematics are as similar as possible.

The prediction error, $e_{pred}\left[k_{s}\right]$, at time-step $k_{s}$ is presented as

(17a) $$\begin{array}{cc} e_{pred}\left[k_{s}\right] & =F_{m}\left[k_{m}\right]-F_{s}\left[k_{s}\right] \end{array}$$

for

(17b) $$\begin{array}{cc} k_{m} & <k_{s} \end{array}$$

(17c) $$\begin{array}{cc} x_{m}\left[k_{m}\right] & \approx x_{s}\left[k_{s}\right] \end{array}$$

(17d) $$\begin{array}{cc} sgn({\dot{x}}_{s}\left[k_{s}\right]) & =sgn({\dot{x}}_{m}\left[k_{m}\right]). \end{array}$$

This is accomplished by:

- Considering the slave position ($x_{s}$) and force ($F_{s}$) at each time-step ($k_{s}$), and using these as the slave reference;
- Searching through the dataset of the master positions, backwards from the slave reference time-step, for the first closest master position ($x_{m}$) to $x_{s}$.
- Comparing the direction of motion of the slave and master to ensure the two systems were travelling in the same direction. If the directions differ, then the next closest slave and master positions are used.
- The time-step when the positions are closest, and the direction of travel is consistent, is $k_{m}$, and is used as the master reference;
- Finding the error between the slave force ($F_{s}\left[k_{s}\right]$) and the master force ($F_{m}\left[k_{m}\right]$) at their respective reference time-steps.

The above method attempts to determine how accurate the predicted force *was* once the slave has passed through the previous master position; the error analysis is *aposteriori*, as opposed to current.

## 4. Experiments

### 4.1. Experimental Setup

**Figure 3:**
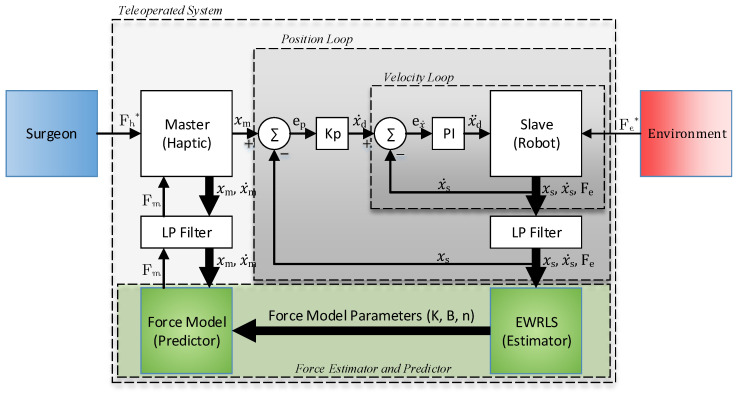
Proposed teleoperator controller.

The teleoperated control structure is shown in Figure 3. The custom control software was written in C++ and runs at 200 Hz on Windows 10, including processes for parameter estimation and force prediction. The system features an outer position P loop and an inner velocity proportional-integral PI loop. The inner velocity loop is internal to the joint motors controller. The position loop gain $K_{p}$ has been tuned to have a minimum rise time with no overshoot. The slave mechanism also has a motion bandwidth of 1 HZ, which is necessary to minimise the effect of the lowest natural frequency of the mechanism at 14 HZ. Low-pass filters (LP) smooth each of the signals. A first-order Butterworth filter with a cut-off frequency of 5 Hz was used to filter position, velocity, and force data for both the slave robot and the master device.

**Figure 4:**
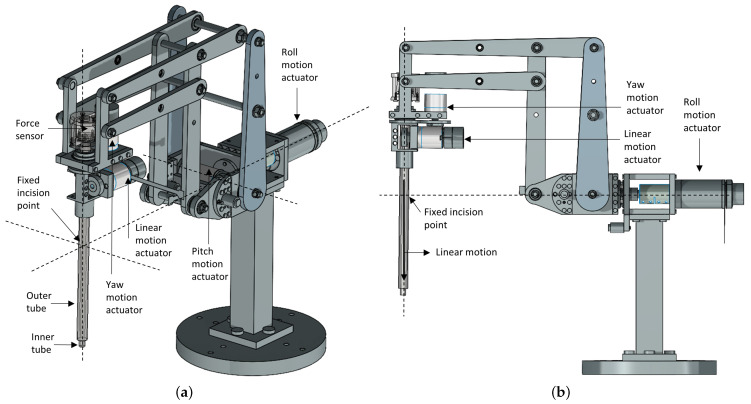
Slave robot with double parallelogram remote-centre-of-motion mechanism design. (**a**) The robot modules and the fixed incision point at the intersection of the robot’s axes of motion. (**b**) Side view of the robot.

The robotic slave is shown in Figure 4. The RMIS procedure relies on the use of a constrained fixed point in the robot workspace in order to minimize undue trauma to the patient. This fixed point is positioned at the point of the patient’s incision. The slave robot features a double parallelogram design, which has a mechanically constrained remote centre-of-motion (RCM) [54,55]. Through an RCM mechanism, robot axes of motion intersect at a fixed point to prevent trauma to the abdominal wall of the patient. The robot has four DOF; three rotations (roll, pitch, yaw) about the fixed incision point and one translation (along the tool axis, driven by friction drive) through the incision point. Maxon EC motors and 4096 count/rev encoders actuate the robot via Maxon EPOS2 24/2 motor controllers, which also provide slave position and velocity signals. The CAN-bus protocol was used to send robot control commands to the four Maxon EPOS2 controllers and read the slave joint data.

**Figure 5:**
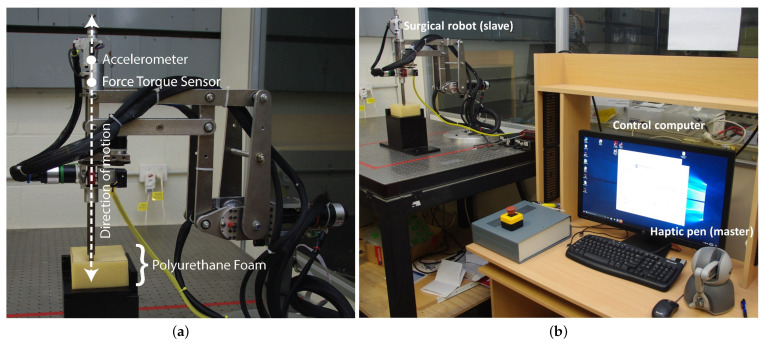
Experimental research facility. (**a**) Surgical robot (slave) in contact with the polyurethane foam. (**b**) Full experimental teleoperated system featuring surgical robot (slave), haptic pen (master), and control computer.

The slave robot linear stage includes a force–torque sensor fixed to the proximal tip of an inner tube Figure 4a. In order to isolate the force–torque sensor from the trocar forces, the inner tube passes through an outer tube, or overcoat, and then is passed through the patient’s incision. The force–torque sensor is a six DOF Nano25 (ATI Industrial Automation) located along the end-effector axis, and an accelerometer is used to compensate for the measured forces of gravity [56]. A Labjack with a USB interface was used to transmit analogue data, including accelerometer readings, to the PC. The end effector is the distal tip of a metal rod positioned perpendicular to the face of the contact material, as shown in Figure 5a.

A picture of the full teleoperated system is shown in Figure 5b. A Phantom Omni haptic feedback device is used as the master input, capable of position forward and force feedback control. Through the standard IEEE-1394 FireWire communication protocol, Phantom Omni’s haptic data, such as position, velocity, and force feedback, were read and sent every cycle. The Cartesian coordinates of the Phantom Omni stylus tip were mapped to robot end-effector tip cartesian coordinates. The specific pose of the Omni was ignored with the exception of a rotation around the stylus’s axis, which was mapped to the rotation around the slave’s linear axis of motion. Feedback forces were limited to 3 N, which is within the Phantom Omni capabilities. Care was also taken to ensure environmental forces at the slave were kept below this level.

### 4.2. Experimental Procedure

The experimental procedure was consistent throughout all the tests performed. First, the surgical slave (controlled via the user-operated master) was gradually pressed perpendicularly into a soft polyurethane foam until an environment force of approximately 3 N was observed. From there, the slave oscillated up and down while in contact with the foam for several tens of seconds until fully retracted from the material. In the current stage of the experiments, force prediction and feedback have been limited to one DOF. Therefore, only the Omni’s *z*-position was mapped to the slave, and since the *x* and *y* coordinates of the Omni were ignored, there was no need for the subject to follow a perfectly linear trajectory using the master stylus. It should also be noted that the slave was only "engaged" to follow the Omni when a button on the haptic stylus was pressed. In addition to the physical force feedback provided by the Phantom Omni stylus, the feedback force was monitored graphically in the GUI to ensure that both the master and the slave remained in contact with the respective real and virtual environments. This procedure was repeated for three trials of varying length and teleoperation speed.

Initial values for the EWRLS estimator were kept constant for each experiment and are listed in Table 2.

Experiments were conducted using:

- Direct force feedback from the slave to the master;
- A spring-based adaptor acting between the slave and master;
- Virtual environments from the EWRLS estimation–prediction methodology, with both the Kelvin–Voigt and Hunt–Crossley force models.

## 5. Results and Discussion

### 5.1. Direct Force Feedback

**Figure 6:**
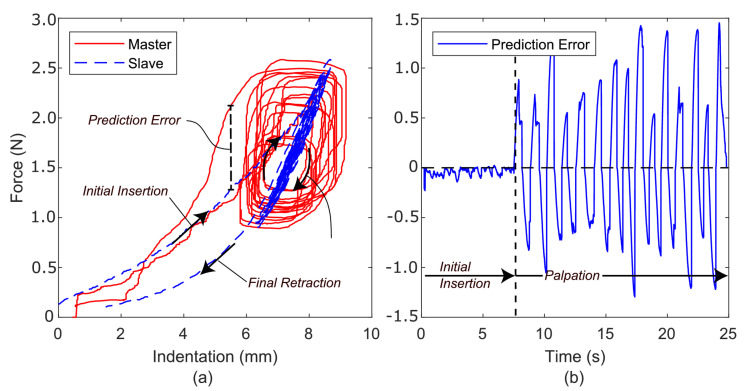
(**a**) Force profiles for both the slave and master as the slave interacts with polyurethane foam using a direct force-feedback channel. (**b**) Prediction error.

Figure 6a compares the slave-environment force with the master-feedback force as the slave interacts with the polyurethane foam. In this experiment, the Butterworth filter cut-off frequency was set to 3 HZ on the $F_{e}$ signal to reduce unwanted noise in the force-feedback signal, $F_{m}\left[k\right]$. No other signals were filtered. During teleoperation, a distinct feeling of delay in the force response could be felt as the environment was manipulated. This perceived delay is seen in the widening of the master force profile. Figure 6b shows the prediction error between the slave and master. While, in this case, it is not strictly a ‘prediction error’, as no prediction has occurred, it is labelled as such due to it being calculated in the manner detailed above in Section 3.1. Distinct components are the *initial insertion*, *palpation*, and *retraction* phases. This figure demonstrates that prediction error increases significantly once palpation begins, ranging between ±1.5 N.

**Figure 7:**
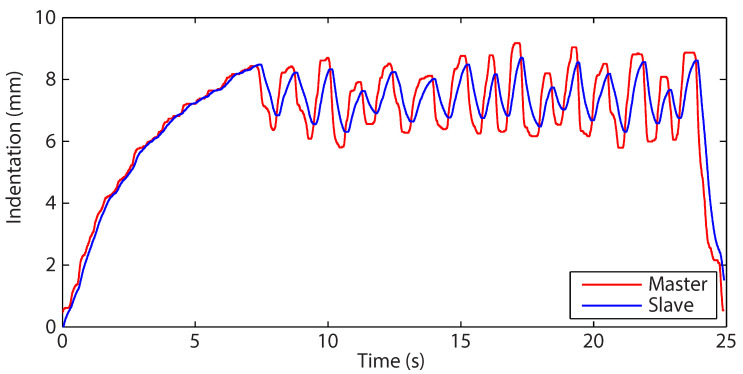
Position profiles for both the slave and master as the slave interacts with the polyurethane foam using the direct force-feedback channel.

The position trajectories of the master and slave are presented in Figure 7. During palpation (from approximately 7.5 s onwards), the slave loses position tracking cohesion with the master. The master can be seen to lead the slave considerably as the system is manipulated against the environment. Force feedback, originating in this experiment *directly* from the measured slave force, is intrinsically tied to the slave position. When there is a substantial offset between the slave and master positions, poor transparency follows.

The root-mean-squared error (RMSE) and mean prediction error during palpation for the three trials are given in Table 3. Prediction error during the initial insertion has been excluded from the calculations because (a) the error is small during this phase, (b) the error during this phase does not give an indication of the teleoperation performance, and (c) the length of time taken to reach full insertion differs considerably across each trial.

The variation in RMSE seen across each trial is due to different teleoperation speeds used while controlling the system. For example, Trial 1 had comparatively faster motions of greater displacement when compared to Trials 2 and 3, which resulted in greater feedback error.

### 5.2. Spring-Based Adaptor

**Figure 8:**
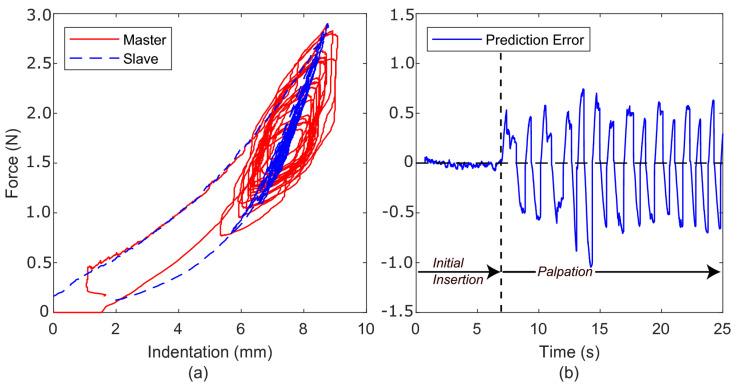
(**a**) Force-indentation profile using the original spring-based adaptor to moderate the feedback force to the master. (**b**) Prediction error during palpation.

Figure 8a shows the force-indentation profile for the slave and the master, where the spring-based adaptor is used to moderate the feedback force. A low-pass filter was used in the calculation of $\hat{K}$ to smooth the noise within the slave force and position signals. Similarly, a cut-off frequency of 3 HZ was applied to the slave force signal $F_{s}$. A maximum value of 0.5 N m${}^{-1}$ for $\hat{K}$ was also specified due to the inaccuracy of the adaptor (10) when the indentation is small ($\left|x_{s}-x_{contact}\right|\ll 1$). The adaptor introduces a restoring force to the master, which acts to pull the master feedback force towards the slave-environment force. As the adaptor takes into account the master position, the feedback force is no longer directly tied to the slave dynamics. Instead, the feedback force is adapted to the environment’s properties and the master’s own kinematics. Figure 8b shows the prediction error between the master’s feedback force and the measured slave-environment force. Similar to Figure 6b, the error is minimal during the initial insertion (0–7 s) but grows once palpation begins.

**Figure 9:**
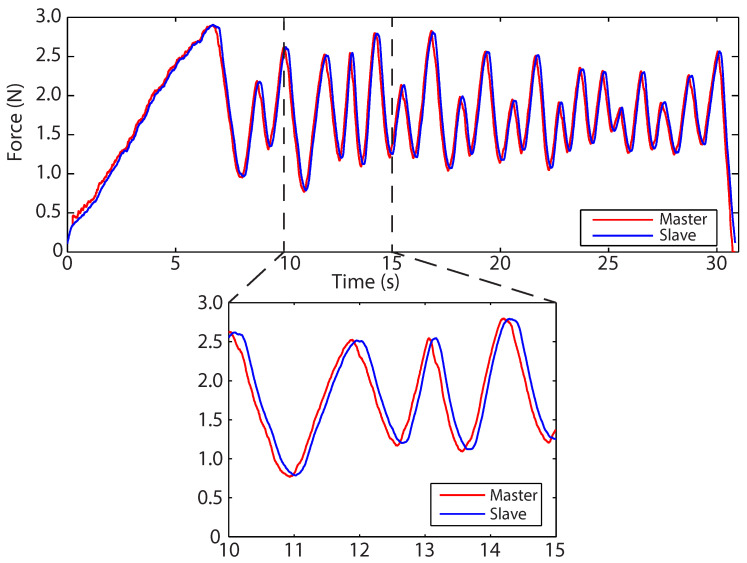
Force–time profiles of the slave and master, using the original spring-based adaptor, with a section highlighting the adaptor acting on the master feedback force.

Figure 9 demonstrates the restoring force acting on the master, effectively pre-empting the changes in force feedback required by the master’s own kinematics. The highlighted section in Figure 9 shows clearly that the force feedback now leads (and predicts) the slave-environment force. Considering that during teleoperation the master similarly leads the slave to position (Figure 7), this effect is greatly desired and improves both the stability and telepresence of the surgical system.

RMSE and mean prediction error for each trial of this experiment are given below in Table 4. Again, the error during the initial insertion is excluded from both the RMSE and mean calculations for a more appropriate comparison between trials. These values are a noticeable improvement over the direct-force feedback methodology.

### 5.3. Kelvin–Voigt Model

**Figure 10:**
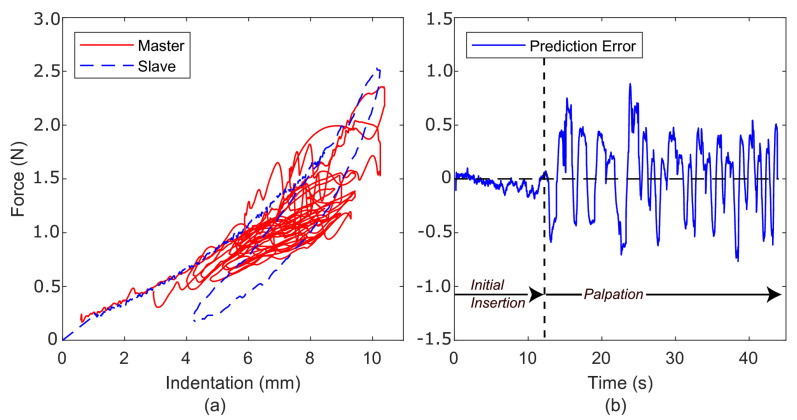
(**a**) Force-indentation profile using the Kelvin–Voigt force model as the virtual environment. Note that only the slave force outer envelope is presented for clarity. (**b**) The master prediction error.

Figure 10a shows the position-force profile for the measured slave-environment force and the predicted master feedback force. For the purpose of clarity, only the outer envelope of the slave force profile is shown. During this experiment, all dynamic signals (position, velocity, and force) were filtered for both the slave and master at a cut-off frequency of 5 HZ. The full form of the slave position-force profile is similar to that in Figure 8. This figure demonstrates that the Kelvin–Voigt model performs poorly when estimating the force response of the polyurethane foam. The Kelvin–Voigt model, being a linear model, is unable to capture the non-linear behaviour of the foam. This is indicated by the overall linear profile of the master feedback force. Additionally, during teleoperation, the estimated parameters $K_{KV}$ and $B_{KV}$ were unable to converge and settle to a satisfactory constant value. Instead, the parameters would vary with each movement of the slave mechanism. This variation is a direct result of the linear Kelvin–Voigt model attempting to capture the material’s restitution, hysteresis, and non-linear effects. The culmination of this is a non-constant, potentially unstable virtual environment.

**Figure 11:**
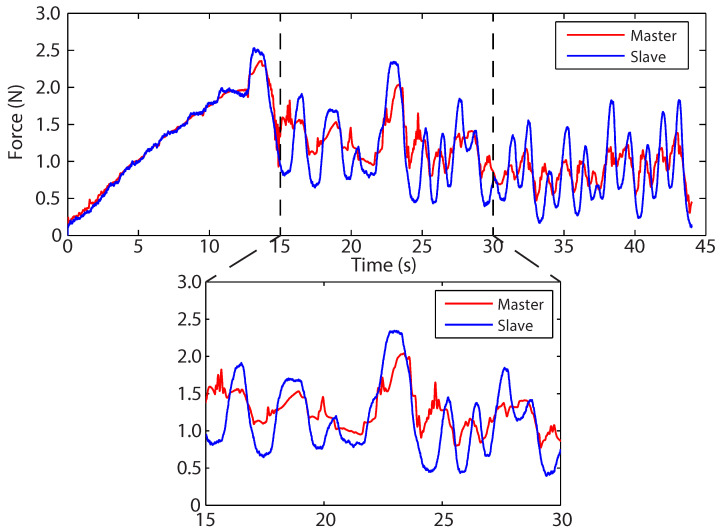
Force–time profile using the Kelvin–Voigt force model as the virtual environment. The highlighted section demonstrates how the master feedback force inconsistently follows the slave environment force.

The prediction error seen in Figure 10b demonstrates a similarly inconsistent performance. When compared with prediction errors for the direct and spring-based methodologies, the virtual Kelvin–Voigt model is more unpredictable and has greater localised variations, with several instances of high-frequency error (≈5 HZ). Further, Figure 11 shows that the predicted feedback force no longer faithfully pre-empts the slave-environment force.

**Figure 12:**
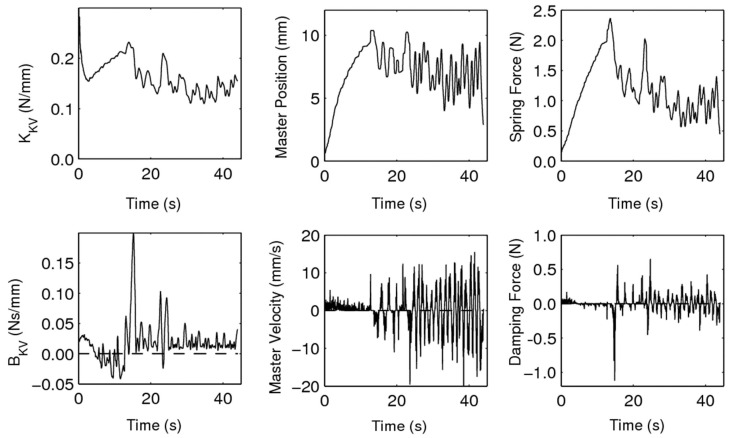
Individual spring and damping force components of the Kelvin–Voigt environment model.

The damping coefficient, $B_{KV}$, was observed to vary noticeably, even becoming negative for portions of the experiment. A negative damping coefficient leads to potentially severely unstable behaviour. This large variation of the damping coefficient, combined with the irregular nature of the PHANToM Omni velocity signal, creates a particularly non-smooth damping force, as per Figure 12.

The RMSE and mean prediction error for each of the three trials is presented in Table 5. In addition, information regarding the movement speed and range is given, with the aim of providing insight into the varied teleoperation performance.

Similar to the direct-feedback methodology, the variation seen in the RMSE values above is due to the different speed and range of motions used during each trial, a consequence of human-controlled teleoperation. Trial 2 had the highest average speed of motion (3.9 mm s${}^{-1}$) and, subsequently, the greatest RMSE (and poorest teleoperation transparency). For comparison, the average speed of Trials 1 and 3 were 2.4 mm s${}^{-1}$ and 2.2 mm s${}^{-1}$, respectively. Additionally, Trial 2 has a significant mean error, which indicates that there was a bias present in either the motion tracking or force measurements taken during this trial, most likely due to the faster-than-expected motions.

Trial 1, which has the lowest RMSE of the three trials, had a more restricted motion range compared with the subsequent trials. The maximum environment force recorded for this trial was 2.51 N. Trials 2 and 3 had maximum forces of 3.31 N and 3.40 N, respectively. The Kelvin–Voigt estimator is less effective over larger ranges of motion as the polyurethane foam’s non-linearity becomes more pronounced. As such, the Kelvin–Voigt model is more accurate during Trial 1, as evident by the considerably lower RMSE.

Overall, the virtual Kelvin–Voigt model demonstrates worse transparency for the polyurethane foam than the spring-based adaptor. However, when the motion range is more constricted and slower, as seen in Trial 1, transparency is slightly improved.

### 5.4. Hunt–Crossley Model

**Figure 13:**
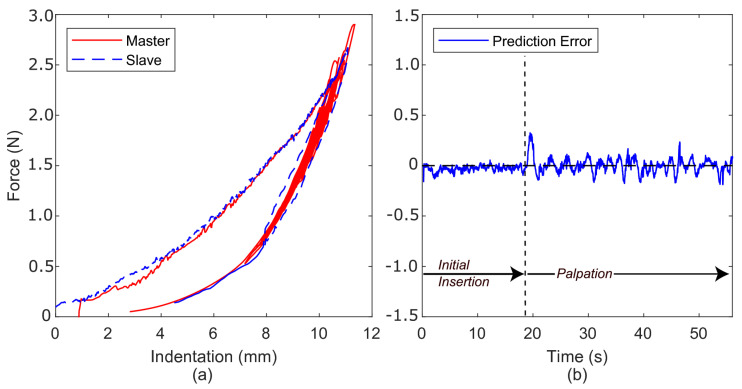
(**a**) Force-indentation profile using the Hunt–Crossley force model as the virtual environment. Note that only the slave force outer envelope is presented for clarity. (**b**) The force-feedback prediction error.

Figure 13a demonstrates the predicted master force-feedback profile closely matching the measured slave-environment profile. As with the previous virtual environment experiment, each position, velocity, and force signal was filtered to 5 Hz with a first-order Butterworth filter. The non-linear constant *n* is able to capture the behaviour of the polyurethane foam, and the master force profile is well contained within the slave profile. Additionally, the position-dependent damping term removes the non-zero force at x(t)=0 present in the Kelvin–Voigt model and spring-based adaptor. The force, during retraction, gradually decreases to zero as the tool is removed from the environment. Figure 13b shows how the prediction error remains low during the experiment, ranging for the most part between ±0.15 N. There is a major peak at approximately 20 s, which was the time at which the slave end-effector transitioned from insertion to palpation.

**Figure 14:**
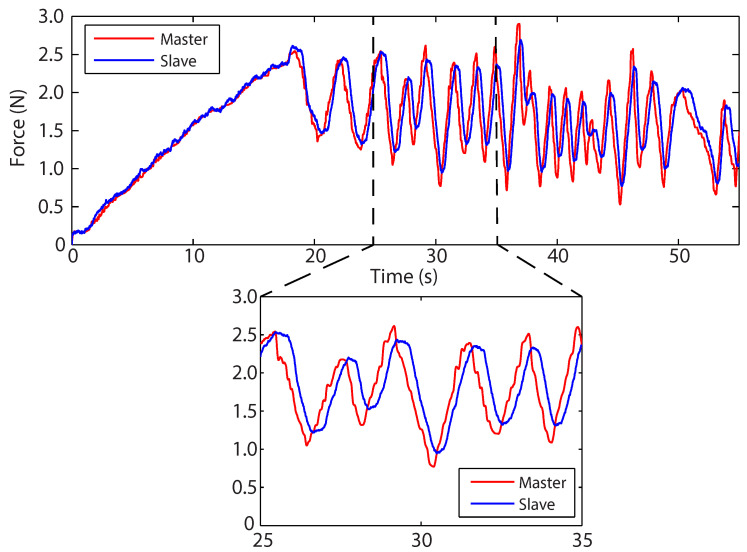
Force–time profile using the Hunt–Crossley force model as the virtual environment. The highlighted section demonstrates the model’s ability to accurately pre-empt the feedback force.

**Figure 15:**
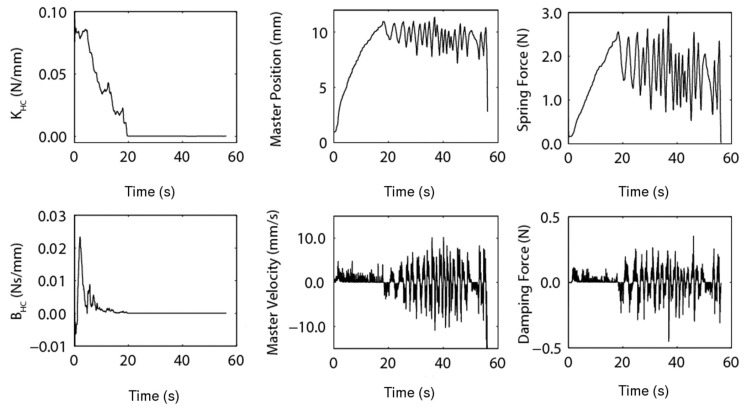
Individual spring and damping force components of the Hunt–Crossley environment model.

Figure 14 demonstrates the Hunt–Crossley virtual environment anticipating the feedback force with the kinematics of the master, effectively causing the master feedback force to lead the slave-environment force. Figure 15 illustrates that the $K_{HC}$ and $B_{HC}$ converged and settled for satisfactory constant values.

The RMSE and mean prediction error for the three trials are given below in Table 6. As with the previous experiments, the RMSE and mean prediction error calculations are performed for the palpation phase. The mean motion speed and maximum environmental force is also referenced. The RMSE of the environment prediction was notably low and consistent across each trial. Trial 2 had the highest RMSE at 0.115 N, and Trial 1 had the lowest at 0.076 N, indicating that the Hunt–Crossley model is capable of predicting the environment to a high degree of accuracy. The mean error was near zero for each trial, demonstrating no observable bias towards any particular direction of motion. The mean speed of motion and maximum environment forces were consistent across each trial, aided, in part, by the relatively high transparency seen during the experiment. Higher transparency meant that, during the teleoperation, there was a greater sense of control over both the robotic slave and haptic master.

**Figure 16:**
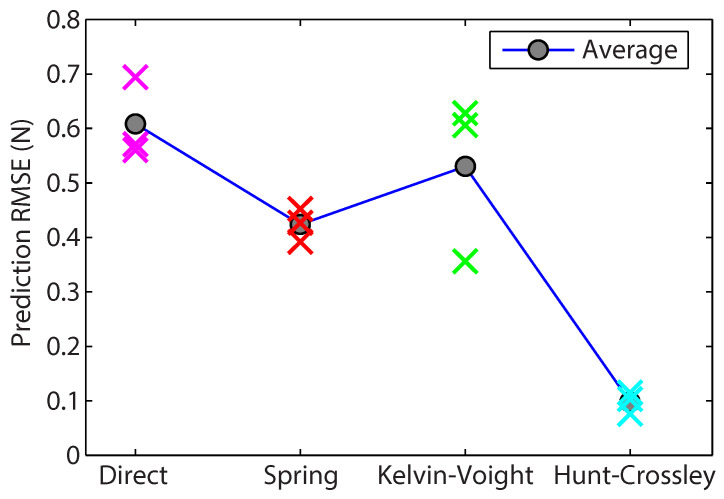
Prediction RMSE for each of the force feedback methodologies tested.

Teleoperator performance was evaluated by analysing the prediction error, the results of which are presented in Figure 16. These results demonstrated that the Hunt–Crossley force model was able to provide greatly improved transparency when compared to the three other force-feedback methods, having an average prediction RMSE error of 0.10 N. Using the Kelvin–Voigt force model exhibited potentially improved performance (Trial 1), but also demonstrated a notable inconsistency between the individual trials. The spring-based adaptor was more consistent but had a higher prediction RMSE of 0.43 N. Using a direct force feedback methodology resulted in considerable prediction error, with an average RMSE of 0.60 N.

## 6. Discussion and Conclusions

In this paper, an environmental estimation and force-predictive bilateral controller were presented using an experimental investigation with a human-in-the-loop palpation task. The controller uses an EWRLS estimator at the slave to generate an estimation of the environment parameters. Force feedback at the master is then predicted using these estimated parameters. By replacing the force-feedback channel with the estimated parameters, issues related to communication time delay between the two subsystems can be alleviated. The experimental results demonstrated significant improvements in teleoperator transparency when the environment is modelled on the non-linear Hunt–Crossley force model.

There are a number of extensions that can be made to this study. Future work will focus on improving the speed of convergence and parameter stability within the environment estimator. The robotic platform is designed to perform more complex four DOF motions with the potential of articulating the end-effector for additional DOFs, but experimental verification was limited to a single DOF at this stage of the study. Future research aims to expand the system to its full four-DOF capabilities. There is also a possibility that the results may be affected by the extent to which the subject is familiar with the teleoperation system. In this study, experiments were conducted by a subject familiar with the robotic system. This was a validation and verification of the teleoperation architecture aimed at confirming whether the estimation–prediction models could be successful in achieving transparency. In a future study, the learning curve of the teleoperation task and the transparency results of users with different levels of familiarity with the system will be evaluated. Furthermore, the experimental verification conducted in this paper was aimed at determining the performance of a single force-feedback model within a single homogeneous soft environment. Studies will be conducted in the future that examine uneven environments with different rigidities at different sections. The study of predictive force feedback with flexible end-effectors would also be a potential future direction for this research. Finally, using vibrotactile feedback [57] can improve the surgeon’s perception of the remote environment, and this could be a possible area for future research.

## Figures and Tables

**Table 1:** Linearised Force Models Used in the EWRLS Algorithm.

	Kelvin–Voigt	Hunt–Crossley
$y_{k}$	$\left[F_{k}\right]$	$\left[\ln(F_{k})\right]$
$\phi_{k}^{T}$	$\left[x_{k},{\dot{x}}_{k}\right]$	$\left[1,{\dot{x}}_{k},\ln\left(x_{k}\right)\right]$
$\theta_{k}$	${\left[K_{KV},B_{KV}\right]}^{T}$	${\left[\ln\left(K_{HC}\right),\frac{B_{HC}}{K_{HC}},n\right]}^{T}$

**Table 2:** Initial Values for the Estimation Algorithm.

Parameter	Initial Value
*Environment Parameters*	
*K*	0.05 N mm${}^{-1}$
*B*	0.01 N s mm${}^{-1}$
*n*	2.00
*EWRLS Algorithm*	
$P_{0}$	*I*
λ	0.995

**Table 3:** RMSE and Mean Prediction Error During Palpation Using a Direct Force Feedback Methodology. Asterisk (*) indicates presented experimental results.

	Trial 1 (*)	Trial 2	Trial 3
RMSE	0.694 N	0.571 N	0.560 N
Mean Error	0.060 N	0.036 N	−0.106 N

**Table 4:** RMSE and mean prediction error during palpation using the spring-based adaptor. Asterisks (*) denote presented data.

	Trial 1 (*)	Trial 2	Trial 3
RMSE	0.427 N	0.452 N	0.392 N
Mean Error	−0.026 N	−0.066 N	0.005 N

**Table 5:** RMSE and mean prediction error during palpation using the Kelvin–Voigt virtual environment. Asterisks denote presented experimental data.

	Trial 1 (*)	Trial 2	Trial 3
RMSE	0.356 N	0.628 N	0.606 N
Mean Error	0.060 N	0.122 N	0.087 N
Mean Speed of Motion	2.4 mm s${}^{-1}$	3.9 mm s${}^{-1}$	2.2 mm s${}^{-1}$
Max. Environmental Force	2.51 N	3.31 N	3.40 N

**Table 6:** RMSE and mean prediction error during palpation using the Hunt–Crossley virtual environment.

	Trial 1	Trial 2	Trial 3
RMSE	0.076 N	0.115 N	0.103 N
Mean Error	0.002 N	0.034 N	−0.002 N
Mean Speed of Motion	1.50 mm s${}^{-1}$	1.55 mm s${}^{-1}$	2.24 mm s${}^{-1}$
Max. Environmental Force	2.69 N	2.30 N	2.48 N

## Data Availability

Not applicable.
